# Surgical Reconstruction with Tendon Allografting Following Iatrogenic Rupture of the Plantar Fascia: A Case Report

**DOI:** 10.3390/medicina58081075

**Published:** 2022-08-10

**Authors:** Chien-Ming Chin, Huan-Ming Tang, Kai-Chiang Yang, Ing-Ho Chen, Chen-Chie Wang

**Affiliations:** 1 Department of Orthopedics, Hualien Tzu Chi Hospital, Tzu Chi University, Buddhist Tzu Chi Medical Foundation, Hualien 970473, Taiwan; 2 Department of Orthopedic Surgery, Dalin Tzu Chi Hospital, Buddhist Tzu Chi Medical Foundation, Chiayi 62247, Taiwan; 3 Department of Orthopedic Surgery, Taipei Tzu Chi Hospital, Buddhist Tzu Chi Medical Foundation, New Taipei City 23142, Taiwan; 4 School of Dental Technology, College of Oral Medicine, Taipei Medical University, Taipei 11031, Taiwan; 5 Department of Orthopedics, School of Medicine, Tzu Chi University, Hualien 97004, Taiwan

**Keywords:** chronic plantar fascia rupture, tendon allograft, Pulvertaft

## Abstract

Plantar fasciitis is a common cause of heel pain, and the disorder is generally self-limiting after adequate conservative treatment. When conservative treatment is unsuccessful, surgical release is an effective treatment option. Here we report a case of iatrogenic plantar fascia rupture after surgical release for treatment of recalcitrant plantar fasciitis. Preoperative MRI revealed a 4.2 cm gap between the distal fascia stump and the calcaneal tuberosity in the sagittal view at 8 months post-injury. To circumvent the possibility of rupture site retear or poor tissue healing by direct repair, we used tendon allografting for the reconstruction of the chronic plantar fascia rupture. The patient gradually recovered after the surgery. Complications of plantar fascia rupture after surgical release is a potential risk but rarely observed. Chronic plantar fascia rupture with medial arch collapse is difficult to treat. We used a tendon allograft to reconstruct the plantar fascia, restoring its function and mechanical strength. After 5 years of follow-up, no complications were reported, and magnetic resonance imaging indicated the reconstructed plantar fascia tissue to be in good condition.

## 1. Introduction

Plantar fasciitis is a highly common cause of heel pain. The proposed etiologies are overload and overuse. Conservative treatments include physical therapy, use of a heel cushion or insole, and night splinting. Local steroid injection and prolotherapy are also indicated as alternative treatments [1,2]. Surgical intervention is considered for patients whose condition does not improve following conservative treatment. Partial plantar fascia release is a practicable option for recalcitrant plantar fasciitis.

However, excessive release may lead to complications such as lateral column syndrome, medial arch collapse, and even iatrogenic plantar fascia rupture [3,4]. Plantar fascia rupture can be divided into acute and acute-on-chronic rupture [5]. For high-demand athletes with acute plantar fascia rupture, functional outcomes can be achieved with surgical repair [6]. However, few studies have investigated the management of chronic ruptures. Following an Achilles tendon rupture, the soft tissue contracts 3–4 days postinjury [7]. In cases of chronic rupture, it is difficult to achieve end-to-end apposition of the tendon ends with plantarflexion of the ankle. Treatments for chronic Achilles tendon rupture, including tendon autografting or allograft reconstruction, have yielded favorable results and adequate functional outcomes [8,9].

Herein, we report an iatrogenic total plantar fascia rupture reconstructed with tendon allografting. After a 5-year follow-up, the patient’s American Orthopedic Foot and Ankle Society (AOFAS) score was 85 with obvious improvement, and magnetic resonance imaging (MRI) findings indicated that his reconstructed plantar fascia was in good condition [10].

## 2. Case Presentation

A 61-year-old obese man (body mass index: 33.45 kg/m^2^) with hypertension under regular control with Amlodipine but otherwise healthy presented to his primary care physician with right heel pain. The pain was located on the bottom of the foot near the posteromedial heel. The patient reported the particular pain when stepping out of bed in the morning or after sitting for a long period. After the diagnosis of plantar fasciitis, he received conservative treatments including physical therapy, shoe modification, and heat pack application. Following the failure of conservative treatments, he underwent open release of the right plantar fascia at a local hospital.

**Figure 1:**
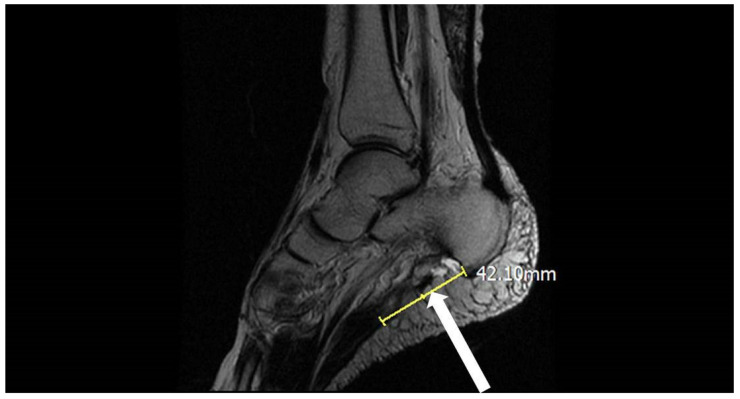
The white arrow indicates the complete rupture of the planta fascia with a 4.2-cm gap between the distal fascia stump and the calcaneal tuberosity on an MRI sagittal view.

Initially, the pain improved. However, 2 weeks postoperatively, he started to feel discomfort accompanied by snapping and popping sensations during ambulation and weight bearing. The type of pain was the same as before, but it was more intense and extended to the posterolateral heel. He sought treatment at multiple hospitals without success. After 8 months of worsening pain following the surgery, he was referred to our orthopedic clinic. The patient also reported a feeling of progressive medial midfoot pain after walking for a long time. A physical examination revealed mild edema and local tenderness near the midline of his heel and superior to the calcaneal tuberosity. When standing, pes planus was apparent. The patient could walk on his toes and heels and had an antalgic gait. The patient’s AOFAS hindfoot score was 54 [10]. MRI revealed the complete rupture of the plantar fascia (see Figure 1) and that the distal fascia stump had a curvilinear shape, indicating a lack of original fascia tension. The sagittal view revealed a 4.2 cm gap between the distal fascia stump and the calcaneal tuberosity. After discussions with the patient and due to concerns about the large fascial gap, we proceeded with the plantar fascia reconstruction with the extensor hallucis longus (EHL) tendon allograft using the Pulvertaft weaving procedure [11].

**Figure 2:**
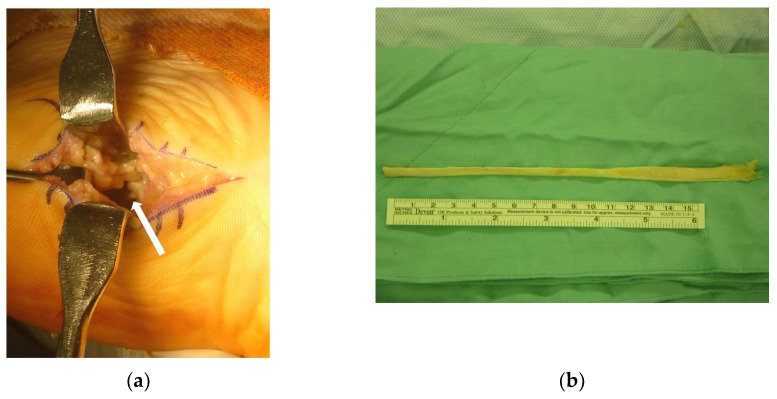
(**a**): The white arrow indicates the ruptured plantar fascia with a 3-cm gap even after extreme plantarflexion. (**b**): The extensor hallucis longus allograft obtained from our bone bank.

During the operation, the ruptured plantar fascia was clearly visible following dissection (see Figure 2a). Large-scale normal irrigation was performed to clear any debris. The ruptured ends of the fascia were then trimmed. The ankle was kept in the plantar-flexed position, and the tendon was approximated. A residual 3 cm gap was noted, and an EHL allograft was then prepared for the fascia reconstruction (see Figure 2b).

**Figure 3:**
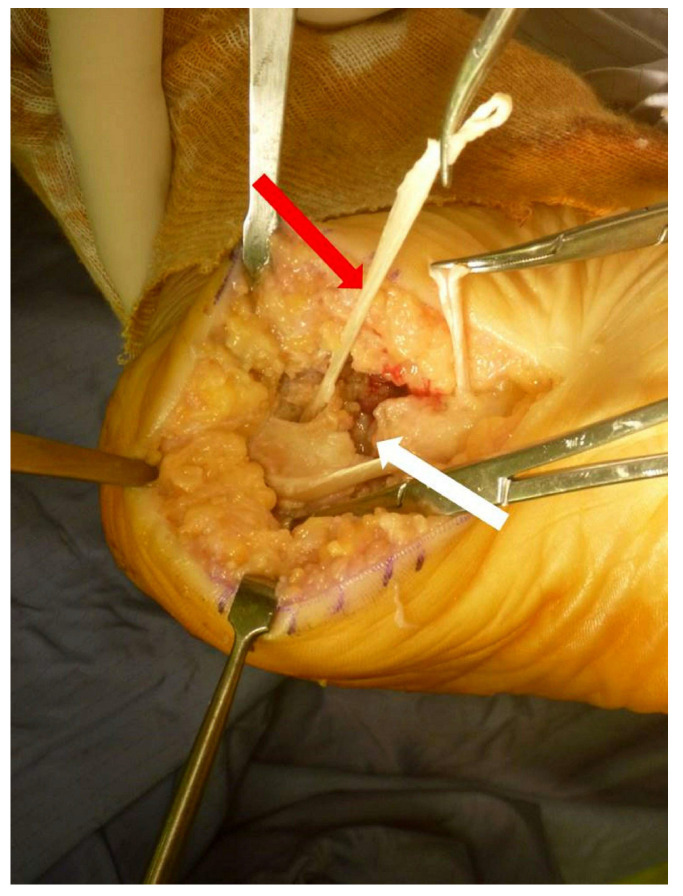
The ruptured fascia was reconstructed with allografting using the Pulvertaft weaving procedure. The white arrow indicates the gap between the two ends, and the red arrow indicates the allograft.

The fascia was reconstructed with a tendon allograft using the Pulvertaft weaving technique (Figure 3).

**Figure 4:**
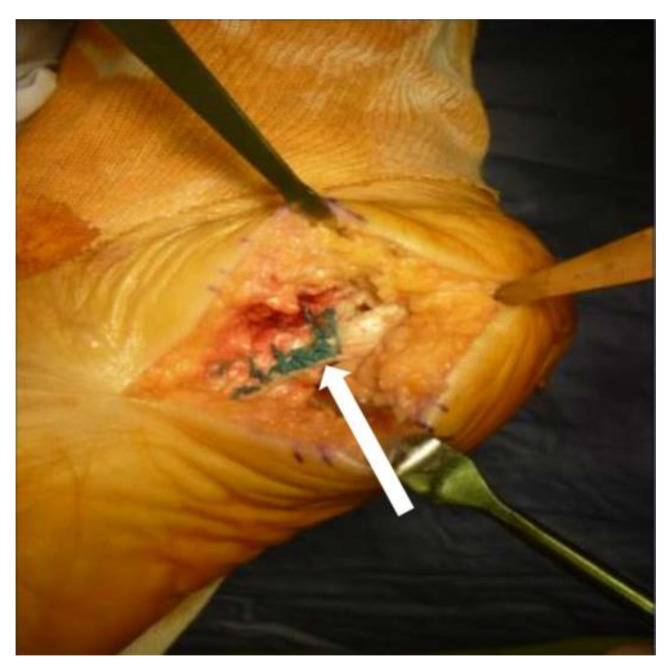
After reconstruction, the 2-0 Ethibond suture was applied to tighten the plantar fascia, as indicated by the white arrow.

A bony tunnel was created near the original area of the plantar fascia. The allograft was passed through the bony tunnel and sutured to the stump of the plantar fascia by Pulvertaft weaving. After we tightened the plantar fascia by pulling the tendon allograft, the whole structure was further secured by 2-0 Ethibond suture (Ethicon, Bridgewater, NJ, USA) (see Figure 4).

**Figure 5:**
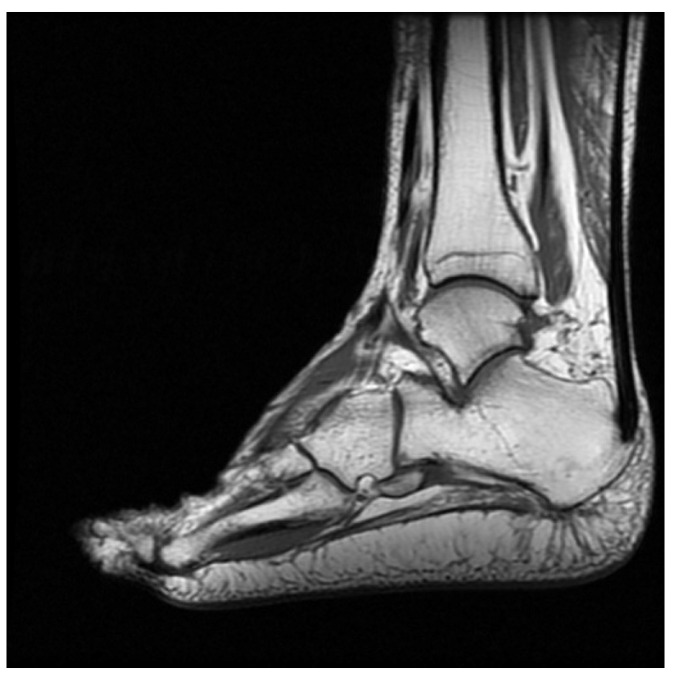
Postoperative MRI shows good tightening of the plantar fascia and the repair of the previous lesion.

A plantar-flexed short leg splint was applied thereafter. Splinting protection was removed 4 weeks postoperatively, and a walking boot was utilized for assistance with walking and partial weight bearing. Full weight bearing was permitted 2 months postoperatively. No complications such as infection were found. Two years after the operation, the patient’s AOFAS score was 85. The allograft was not rejected, and MRI showed good thickness and continuity of the repaired plantar fascia (see Figure 5).

## 3. Discussion

Plantar fasciitis is a common disorder and is the cause of 11% to 15% of cases of foot and ankle discomfort in daily practice [1,2]. Plantar fasciitis is a self-limiting disease, and discomfort symptoms resolve in 80% to 90% of cases within nine months [3]. For those who achieve poor results with conservative treatments, surgical interventions such as gastrocnemius recession or plantar fascia partial release are recommended [3,4]. Potential complications include destabilized medial column, lateral column pain, medial longitudinal arch collapse, plantar nerve injury, complex regional pain syndrome, persistent or recurrent pain, and even iatrogenic complete plantar fascia rupture [3,4].

Plantar fascia rupture is a rare disorder that can be distinguished into acute and acute-on-chronic rupture [5]. The risk factors of plantar fascia rupture are not well established due to most literatures being case reports, but the previous usage of fluoroquinolone, corticosteroid therapy, diabetes, statin usage and hypertension might increase the risk of tendon rupture which may also hazard the architecture of plantar fascia [12,13,14]. Few case reports are found in the literature, and treatment strategies and therapy concepts are varied, including non-weight bearing and immobilization of the affected limbs [15,16,17]. Nonetheless, surgical intervention for plantar fascia rupture, such as repair or reconstruction, is rarely mentioned. Susanne et al. reported that in high-demand athletes, the surgical repair of a complete plantar fascia rupture with suture anchors or nonabsorbable suture string can provide sufficient mechanical strength and that the patient can return to their previous activity level within 12 months [6].

However, this type of repair should be undertaken on the reparable fascia tissue of the distal stump with no obvious fascial gap between the calcaneal tuberosity and distal fascia stump. In this case, however, we noted a 3 cm gap between the ruptured end and the calcaneal tuberosity even after plantarflexion. This condition made repair challenging. Overtightening of a ruptured fascia end to ensure direct contact of the stump with the calcaneal insertional site can increase the risk of fascia re-rupture or poor tissue healing. Because the result was unpredictable, we chose a tendon allograft for the plantar fascia reconstruction. Although no study has reported on the reconstruction of a plantar fascia rupture with a tendon allograft, studies have reported that allograft reconstructions in chronic Achilles tendon ruptures and quadriceps tendon ruptures yield good clinical outcomes [8,18]. An allograft not only provides sufficient mechanical strength to restore the plantar fascia but also has superior biological properties compared with suture anchors or nonabsorbable suture materials. In addition, when the tendon autograft was chosen, the use of the allograft prevented donor site morbidity. However, any allograft carries the potential risk of disease transmission and graft rejection. Although several studies have reported a low risk of viral disease transmission, a report by the American Association of Tissue Banks indicated no reported incidents of viral disease in over two million cases of orthopedic bone and tendon allografts [19,20,21]. In this case, no infections or wound complications were noted. After a 5-year follow-up, the patient had an AOFAS score of 85, exhibited obvious improvement, had no rejection of the allograft, and had MRI findings showing that the reconstructed plantar fascia was in good condition. Herein, we recommend the usage of tendon allografts with the Pulvertaft weaving procedure to reconstruct chronic plantar fascia ruptures.

## Data Availability

The authors confirm that the data supporting the findings of this study are available within the article.
